# Efficiency analysis of primary health care resources: DEA and Tobit regression evidence from village clinics in Jiangsu Province

**DOI:** 10.3389/fpubh.2025.1515532

**Published:** 2025-04-23

**Authors:** Wei Jiang, Boyi Yang, Xiaoli Dai, Shanshan Li

**Affiliations:** ^1^Department of Medicine, Jiangsu Medical College, Yancheng, China; ^2^Department of Geriatrics, Tongji Hospital, Tongji Medical College, Huazhong University of Science and Technology, Wuhan, China

**Keywords:** primary health care, efficiency, village clinics, data envelopment analysis, Malmquist productivity index, Tobit regression

## Abstract

**Background:**

Village clinics are essential for delivering primary health care in rural China, yet their resource allocation efficiency remains a concern. Many clinics face challenges such as low technical efficiency, imbalanced resource distribution, and insufficient technological progress, which may hinder the delivery of quality healthcare services.

**Methods:**

This study evaluates the resource allocation efficiency of village clinics across 13 cities in Jiangsu Province, China, using Data Envelopment Analysis. The Malmquist Productivity Index was applied to assess efficiency changes over time, and Tobit regression was employed to identify influencing factors.

**Results:**

The overall efficiency of village clinic resource allocation in Jiangsu Province is suboptimal. In 2022, the average technical efficiency was 0.869, with seven cities classified as inefficient. Among them, three exhibited decreasing returns to scale, while four demonstrated increasing returns to scale. Reducing the number of village clinics and health technicians while increasing medical revenue could improve efficiency. From 2015 to 2022, the average Malmquist Productivity Index was 0.96, with a significant decline of 11.6% in 2021–2022, primarily due to a 6.8% decrease in technological change. Random-effects Tobit regression revealed that population density positively correlates with technical efficiency (coefficient = 0.0014, *p* < 0.05), whereas per capita disposable income, healthcare fiscal expenditure, and urbanization rate showed no statistically significant effects.

**Conclusion:**

The resource allocation efficiency of village clinics in Jiangsu Province is insufficient, with technological change being a key driver of efficiency fluctuations. Population density plays a significant role in efficiency variation. To enhance efficiency, optimizing resource allocation strategies and promoting technological advancements are essential for strengthening rural primary health care.

## 1 Introduction

Primary Health Care (PHC) is essential in global health systems, focusing on providing affordable, accessible, and basic health services to the population (1). The 1978 Alma-Ata Declaration advocated for “countries and international organizations to strengthen cooperation and jointly pursue the goals of primary health care, particularly in resource-limited developing countries” (2). Since then, the Chinese government has actively implemented PHC strategies, expediting the achievement of universal coverage (3, 4). Recently, China has made substantial progress in advancing PHC through reforms in its healthcare system (5, 6).

Primary health care institutions are the primary providers of PHC services, responsible for public health tasks such as disease prevention, health education, and maternal and child health (7, 8). As healthcare reform deepens in China, the coverage of PHC services is expanding, especially in rural areas (9). Under the hierarchical diagnosis and treatment system, most diseases are managed at primary health care institutions. However, significant disparities in equipment, staffing, and technical capabilities among these institutions lead to uneven quality in PHC services (10–12). This issue is closely linked to the effective allocation of resources (13–15). First, there is a severe imbalance in health resource distribution between regions, especially between the eastern and western parts of the country, and between urban and rural areas (16–18). Second, inadequate resource allocation in rural primary health care institutions remains a critical issue. In particular, rural primary health care faces numerous challenges that directly affect its service capacity and efficiency, subsequently impacting the quality of care and patient experience. One of the most urgent problems is the shortage of qualified healthcare personnel in rural areas. A qualitative study interviewing 51 village doctors in Shandong Province revealed widespread challenges, including an aging workforce, gender imbalance, low educational attainment, insufficient professional training, heavy workload, and inadequate financial incentives. Additionally, low income, limited social security, and unfair performance evaluation systems have further exacerbated the recruitment and retention crisis in rural healthcare (19). Beyond human resource shortages, accessibility to healthcare services in rural areas remains uneven. A field study conducted in Liannan Yao Autonomous County found that due to transportation and infrastructure constraints, there are significant disparities in the distribution of healthcare resources and the accessibility of services (20). Finally, as the aging population and chronic disease incidence rise, there is an urgent need to optimize resource allocation to improve service efficiency (21–23). Thus, enhancing the efficiency of PHC resource allocation not only improves the quality of medical services but also promotes health equity and sustainable development (24, 25).

China’s primary healthcare system comprises urban community health service centers and stations, as well as rural township health centers and village clinics. The literature assessing the efficiency of primary healthcare resource allocation predominantly merges urban and rural analyses, with some studies examining township health centers in rural areas. However, there is a notable lack of research focusing specifically on village clinics, which serve as the first point of contact for rural residents and are widely distributed. Recent studies have shown a declining trend in the utilization of village clinics. Between 2011 and 2018, the probability of individuals seeking care at village clinics decreased by 44%, while the self-treatment rate among rural residents increased by 20%. This trend suggests that the role of village clinics in China’s rural healthcare system is diminishing, potentially due to resource constraints and low service efficiency (26). Investigating village clinics holds significant theoretical and practical value, as it can yield actionable insights for policymakers to better address the healthcare needs of rural populations (27).

Data Envelopment Analysis (DEA) is a widely used non-parametric method for assessing relative efficiency, particularly in the healthcare sector. Recent studies in China have increasingly utilized DEA to analyze healthcare resource allocation efficiency and explore performance optimization under varying policy contexts (28–30).

This study distinguishes itself by focusing on village clinics across various cities in Jiangsu Province. It employs the DEA model to assess their resource allocation efficiency and integrates the Malmquist productivity index for a dynamic analysis of healthcare resource efficiency. Additionally, Tobit regression analysis will be conducted to identify factors influencing efficiency, revealing potential issues and improvement avenues in the resource utilization of village clinics in Jiangsu Province. This research aims to provide empirical evidence to support the enhancement of healthcare resource allocation and policy optimization.

## 2 Materials and methods

### 2.1 Data sources and variables

This study examines healthcare resources in village clinics across 13 cities in Jiangsu Province. In the healthcare efficiency literature, commonly used input indicators typically encompass three key dimensions: physical resources, human resources, and financial resources. These include variables such as the number of healthcare institutions, hospital beds, health technicians, and medical expenditures. Output indicators generally focus on service utilization and performance outcomes, commonly measured by patient visits, inpatient admissions, and hospital discharges (22, 31, 32). For environmental variables, which help assess the external factors influencing efficiency, prior studies have typically considered economic, social, and policy-related factors. Frequently used environmental variables include fiscal revenue, healthcare fiscal expenditure, per capita GDP, population density, urbanization rate, proportion of urban population, proportion of older adult population, education level, and average years of schooling (33, 34). Based on these established frameworks and considering data availability and representativeness, this study selected the following variables: Inputs: Number of village clinics, number of medical devices, number of health technicians, and total expenditure. These represent the core resource allocation in village clinics.

Outputs: Number of patient visits and medical revenue. These indicators reflect both healthcare service utilization and economic performance. Environmental variables: Per capita disposable income, healthcare fiscal expenditure, population density, and urbanization rate, which account for the broader socioeconomic context influencing efficiency (see Table 1). Data were sourced from the *Jiangsu Health and Family Planning Yearbook* (2016–2019), the *Jiangsu Health Yearbook* (2020–2023), and the *Jiangsu Statistical Yearbook* (2016–2023).

**Table 1:** Variables for the DEA-Tobit model.

Variable		Observations	Mean	Std. dev.	Max	Min
Inputs	Number of village clinics (thousand)	104	1.167	0.761	3.005	0.197
	Number of medical devices (thousand)	104	10.982	18.830	189.088	1.051
	Number of health technicians (thousand)	104	1.325	1.265	4.382	0.008
	Total expenditure (million)	104	241.382	129.764	588.102	33.216
Outputs	Number of patient visits (million)	104	6.462	4.303	17.532	0.860
	Medical revenue (million)	104	157.848	82.408	339.429	28.058
Independent variable	Per capita disposable income (thousand)	104	23.950	7.041	43.785	12.772
	Healthcare fiscal expenditure (billion)	104	6.905	3.813	25.825	2.176
	Population density	104	236.749	52.959	361.292	130.528
	Urbanization rate	104	70.042	8.629	87.000	56.300

### 2.2 Methodology

DEA is a non-parametric method for evaluating the relative efficiency of Decision-Making Units (DMUs) with multiple inputs and outputs (35). It constructs a production frontier and measures each DMU’s distance from this frontier to assess efficiency. DEA is suitable for both input-oriented and output-oriented models (36, 37). The CCR model, developed by Charnes, Cooper, and Rhodes, assumes constant returns to scale (CRS), implying a proportional relationship between input increases and output growth. It calculates technical efficiency (TE), which includes scale efficiency. A TE score of 1 indicates efficiency, while a score below 1 indicates inefficiency. The BCC model, introduced by Banker, Charnes, and Cooper, allows for variable returns to scale (VRS), meaning that outputs may increase at a different rate than inputs (38). The BCC model decomposes efficiency into pure technical efficiency (PTE) and scale efficiency (SE), where PTE measures the efficiency of production technology and SE assesses whether a DMU operates at an optimal scale. The relationship between these metrics is defined as: TE = PTE × SE.

The DEA-Malmquist Productivity Index (MPI) is a tool for analyzing efficiency changes over time (39). It assesses not only efficiency variations but also the impact of technological progress on productivity. For each DMU, the MPI, along with changes in technical efficiency change (TEC), technological change (TC), pure technical efficiency change (PTEC), and scale efficiency change (SEC), can be measured. TEC reflects changes in relative efficiency over time, while TC measures shifts in the production frontier, indicating technological progress. The MPI formula is:

$$MPI=\left(\frac{D_{0}^{t+1}\left(x^{t+1},y^{t+1}\right)}{D_{0}^{t}\left(x^{t},y^{t}\right)}\right)\times {\left(\frac{D_{0}^{t+1}\left(x^{t},y^{t}\right)}{D_{0}^{t}\left(x^{t+1},y^{t+1}\right)}\right)}^{\frac{1}{2}}$$

where $D_{0}^{t}\left(x^{t},y^{t}\right)$ represents the distance function based on the period t production frontier, indicating the efficiency of inputs and outputs. An MPI greater than 1 indicates improved productivity, equal to 1 indicates no change, and less than 1 indicates a decline.

The TE scores range from 0 to 1 and are censored. To address this, we used the Tobit model, a censored regression model based on maximum likelihood estimation (40, 41). For panel data analysis, a random-effects Tobit model was used, selected through a likelihood ratio (LR) test. Considering the panel structure and potential heterogeneity, we employed the random-effects Tobit model. The model is specified as: *θ* = *β_0_ + β_1_X_1_ + β_2_X_2_ + β_3_X_3_ + β_4_X_4_ + ϵ,* where *θ* represents the dependent variable, *X_1_* to *X_4_* are the four environmental variables, and *ϵ* is the error term. Given the relatively small sample size, we employed the Bootstrap method to enhance the robustness and reliability of the TE estimates before conducting the Tobit regression analysis.

Internal Validity was conducted by systematically excluding individual input and output variables to assess their impact on efficiency scores. Spearman correlation analysis evaluated ranking consistency, and Wilcoxon signed-rank tests assessed efficiency changes. External Validity was examined by comparing efficiency score distributions across consecutive years. DEA efficiency scores were validated against Stochastic Frontier Analysis (SFA) estimates to assess consistency.

The DEA-BCC model and MPI were calculated using DEAP 2.1, while SFA was performed in R 4.2.2. Bootstrap resampling and Tobit regression analysis were conducted in Stata 15.0. Statistical significance was set at *p* < 0.05.

## 3 Results

### 3.1 DEA efficiency analysis

In 2022, the TE of village clinics in 13 cities of Jiangsu Province was 0.869, with a PTE of 0.930 and an SE of 0.931. Five cities, accounting for 38.5%, had a TE below the average. Among these, Yangzhou exhibited the lowest TE at 0.514, with a PTE of 0.665 and an SE of 0.773. Wuxi, Changzhou, Suzhou, Nantong, Lianyungang, and Zhenjiang were DEA-efficient, while Nanjing, Xuzhou, Huai’an, Yancheng, Yangzhou, Taizhou, and Suqian were DEA-inefficient. All DEA-inefficient cities exhibited insufficient scale efficiency. Among them, Nanjing, Huai’an, Yangzhou, and Taizhou operated under increasing returns to scale, whereas Xuzhou, Yancheng, and Suqian operated under decreasing returns to scale. Additionally, apart from Nanjing and Xuzhou, the other DEA-inefficient cities also exhibited deficiencies in PTE (Table 2).

**Table 2:** DEA results in 2022.

City	TE	PTE	SE	Type of scale inefficiency
Nanjing	0.662	1	0.662	irs
Wuxi	1	1	1	-
Xuzhou	0.904	1	0.904	drs
Changzhou	1	1	1	-
Suzhou	1	1	1	-
Nantong	1	1	1	-
Lianyungang	1	1	1	-
Huai’an	0.698	0.748	0.934	irs
Yancheng	0.795	0.94	0.846	drs
Yangzhou	0.514	0.665	0.773	irs
Zhenjiang	1	1	1	-
Taizhou	0.741	0.752	0.985	irs
Suqian	0.978	0.984	0.994	drs
Mean	0.869	0.930	0.931	

### 3.2 Input–output redundancy analysis

In DEA-inefficient cities, Nanjing and Xuzhou exhibited no input–output redundancy in their village clinics’ resource allocation. To achieve DEA efficiency, Huai’an would need to reduce its number of village clinics by 127, decrease practicing (assistant) physicians and registered nurses by 250, and increase medical income by 14.556 million. Yancheng should reduce its clinics by 19 and practitioners by 1,398, while increasing medical income by 43.386 million. Yangzhou needs to decrease its number of practicing (assistant) physicians and nurses by 328 and raise medical income by 22.867 million. Taizhou must cut its equipment count by 390, reduce practitioners by 18, and increase medical income by 40.28 million. Lastly, Suqian requires a reduction of 2,209 pieces of equipment and 1,783 practitioners, along with an increase in medical income of 27.006 million (Table 3).

**Table 3:** Input–output redundancy in non-DEA efficient cities, 2022.

City	Output slacks		Input slacks			
	Number of patient visits (million)	Medical revenue (million)	Number of village clinics (thousand)	Number of medical devices (thousand)	Number of health technicians (thousand)	Total expenditure (million)
Nanjing	0.000	0.000	0.000	0.000	0.000	0.000
Xuzhou	0.000	0.000	0.000	0.000	0.000	0.000
Huai’an	0.000	14.556	0.127	0.000	0.250	0.000
Yancheng	0.000	43.386	0.019	0.000	1.398	0.000
Yangzhou	0.000	22.867	0.000	0.000	0.328	0.000
Taizhou	0.000	40.280	0.000	0.390	0.018	0.000
Suqian	0.000	27.006	0.000	2.209	1.783	0.000

### 3.3 MPI analysis across cities

Among the 13 cities in Jiangsu Province, Wuxi, Changzhou, and Suzhou had MPI greater than 1, indicating productivity improvements. Conversely, the MPI for the remaining 10 cities was below 1, signifying a general decline in productivity. Cities with MPI below the average included Nanjing, Huai’an, Yangzhou, Zhenjiang, Taizhou, and Suqian. Yangzhou experienced the most significant decline, with a 10.9% drop in MPI, accompanied by decreases in PTEC, SEC, and TC. Nantong, Lianyungang, and Zhenjiang primarily experienced TC declines, with the most pronounced reductions observed in Nantong and Zhenjiang (Table 4).

**Table 4:** MPI and its decomposition at the provincial level, 2015–2022.

City	EFFCH	TECHCH	PECH	SECH	MPI
Nanjing	0.95	0.986	1	0.95	0.937
Wuxi	1	1.042	1	1	1.042
Xuzhou	0.993	0.976	1	0.993	0.968
Changzhou	1.039	1.001	1.036	1.003	1.04
Suzhou	1	1.002	1	1	1.002
Nantong	1.026	0.954	1.024	1.002	0.979
Lianyungang	1	0.971	1	1	0.971
Huai’an	0.95	0.951	0.959	0.99	0.903
Yancheng	1.007	0.963	1.002	1.005	0.969
Yangzhou	0.926	0.962	0.959	0.966	0.891
Zhenjiang	1	0.954	1	1	0.954
Taizhou	0.958	0.941	0.96	0.998	0.901
Suqian	0.997	0.935	0.998	0.999	0.932
Mean	0.988	0.972	0.995	0.993	0.96

### 3.4 MPI analysis over time

From 2015 to 2022, the average MPI for village clinics in Jiangsu Province was 0.96. The MPI values for 2015–2016 and 2021–2022 were 0.961 and 0.88, reflecting an 8.4% decline. The MPI increased annually from 2015 to 2018, followed by a decrease in 2019, then resumed an upward trend until another significant decline occurred in 2021–2022, with an overall MPI decrease of 11.6%. This decline was characterized by a 4.2% drop in TEC, a 2.6% reduction in SEC, a 1.7% decrease in PTEC, and a 6.8% drop in TC, indicating that TC was the primary factor influencing the MPI of village clinics in Jiangsu Province (Table 5).

**Table 5:** MPI and its decomposition, 2015–2022.

Year	EFFCH	TECHCH	PECH	SECH	MPI
2015–2016	0.993	0.968	1.016	0.978	0.961
2016–2017	0.998	0.983	1.02	0.979	0.982
2017–2018	1.006	0.991	0.994	1.012	0.998
2018–2019	0.967	0.976	0.981	0.986	0.944
2019–2020	1.019	0.944	1	1.019	0.962
2020–2021	0.987	1.009	0.986	1.001	0.996
2021–2022	0.945	0.931	0.969	0.975	0.88
Mean	0.988	0.972	0.995	0.993	0.96

### 3.5 Sensitivity and stability analysis

Internal Validity: The results indicate that removing multiple input and output variables had minimal impact on overall efficiency scores, confirming the internal robustness of the DEA model (Supplementary Table 1). External Validity: The results show a consistent trend in efficiency scores over time, supporting the model’s temporal stability (Supplementary Table 2). The efficiency estimates from SFA were not statistically different from the DEA results, reinforcing the robustness of our findings (Supplementary Table 3).

### 3.6 Tobit regression analysis

The LR test results from the random-effects Tobit model (*p* < 0.001) indicated that this model was more appropriate for the data than the standard Tobit model. The maximum variance inflation factor (VIF) was 3.98, suggesting no multicollinearity among the independent variables. After bootstrapping, the Tobit regression results showed a positive correlation between population density and village clinic TE, with a regression coefficient of 0.0014, which was statistically significant (*p* = 0.002). In contrast, the regression coefficients for per capita disposable income, healthcare fiscal expenditure, and urbanization rate were −0.0014, 0.0036, and −0.0007, respectively, indicating no significant statistical effect on the TE of village clinic resource allocation (*p* > 0.05) (Table 6).

**Table 6:** Tobit regression model results.

Variables	Observed Coef.	Bootstrap Std. Err	*p*-value	VIF	Normal-based [95% Conf. Interval]
Per capita disposable income (thousand)	−0.0014	0.0116	0.901	3.98	[−0.0241, 0.0212]
Healthcare fiscal expenditure (billion)	0.0036	0.0161	0.822	2.96	[−0.0280, 0.0352]
Population density	0.0014	0.0004	0.002	1.84	[0.0005, 0.0022]
Urbanization rate	−0.0007	0.00799	0.930	1.06	[−0.0164, 0.0150]
LR Test	63.99		<0.001		

## 4 Discussion

In rural China, village clinics play a critical role in primary healthcare, offering essential medical services, public health management, and health education. With the advancement of rural healthcare reforms, the efficiency of resource allocation in village clinics has attracted increasing attention from researchers. However, most studies focus on township health centers, while those specifically addressing village clinics remain scarce. Several studies have examined healthcare efficiency across multiple provinces in China. One study assessing rural healthcare resource efficiency in 29 provinces found that economically developed provinces such as Jiangsu, Guangdong, and Zhejiang, despite allocating substantial government healthcare expenditures, were not on the efficiency frontier. In contrast, provinces with a higher proportion of rural populations, such as Hunan, Jiangxi, Henan, and Sichuan, exhibited higher efficiency levels (42). Another study analyzing the efficiency of township health centers in China from 2012 to 2021 found that overall resource allocation efficiency remained relatively low over the decade. Notably, the average efficiency scores for nine provinces, including Jiangsu, were below 0.6, highlighting significant regional disparities in healthcare efficiency across China (43). Comparative studies in other countries provide further insight into primary healthcare efficiency. For instance, an input-oriented bias-corrected DEA analysis of Indian states found an average efficiency score of 0.60, with 14 states scoring below this threshold and 15 above it (44). In Sierra Leone, community health facilities exhibited technical inefficiencies, with efficiency scores ranging from 0.59 to 0.692 (45). Similarly, primary healthcare efficiency varied across Africa, with Nouna, Burkina Faso (0.862) performing better than Zambia (0.619) and Sierra Leone (0.78), though a high DEA score did not necessarily indicate effective management (46). In Ethiopia’s Jimma district, 50% of public health centers were technically inefficient despite an average efficiency of 0.9 (47), while Iran’s Hamadan province saw efficiency scores fluctuate between 0.56 and 0.78 from 2003 to 2013 (48). A broader study of 191 countries found that 78.5% were inefficient in utilizing healthcare resources to achieve universal health coverage (49). These findings suggest that inefficiencies in healthcare resource allocation are a global challenge, not unique to Jiangsu Province.

This study examines the efficiency of healthcare resource allocation in village clinics across 13 cities in Jiangsu Province, China, from 2015 to 2022, and analyzes the factors influencing this efficiency. The findings reveal that in 2022, the overall efficiency of resource allocation in village clinics across Jiangsu Province was suboptimal, with 7 cities identified as DEA-inefficient. Of these, 3 cities had decreasing returns to scale and 4 had increasing returns. From 2015 to 2022, the MPI of village clinics in Jiangsu Province generally declined, driven mainly by decreases in TC. Regression analysis identified population density as a significant influencing factor.

### 4.1 Current status and analysis of resource allocation efficiency

The TE of village clinics in Jiangsu Province was 0.869, indicating widespread inefficiencies in resource allocation, which can undermine service quality and impact the health of rural residents. Multiple factors contribute to this, including technical capacity, scale, management practices, and policies. Six cities achieved DEA efficiency, reflecting high resource utilization, effective management, and service mechanisms. In contrast, seven cities were DEA inefficient. Among these, four cities exhibited increasing returns to scale, implying that their village clinics may be operating below an optimal scale, with insufficient resource investment limiting service capacity. Meanwhile, three cities showed decreasing returns to scale, indicating potential resource redundancy or underutilization, leading to inefficiencies. These findings are consistent with previous research (48). Additionally, five cities exhibited deficiencies in both SE and PTE. This suggests that efficiency disparities among cities in Jiangsu Province stem from both an unbalanced resource supply structure and insufficient internal management and professional operational capacity.

Our results indicate a general decline in the MPI from 2018 to 2022, with a particularly noticeable drop between 2020 and 2022, which may be linked to the COVID-19 pandemic. Several studies suggest that the COVID-19 pandemic significantly disrupted healthcare efficiency worldwide. A study analyzing OECD countries found that lockdowns and quarantine measures did not immediately impact country-level healthcare efficiency; however, delayed lockdowns led to significantly lower efficiency levels during the first COVID-19 wave in 2020 (50). Another study has shown that the efficiency of township health centers in rural China experienced the most significant decline between 2019 and 2020, which has been attributed to the effects of COVID-19 (43). As foundational units of primary healthcare, village clinics likely faced disruptions such as resource reallocation, reduced patient visits, and financial constraints during the pandemic. Additionally, in 2018, the Chinese government issued a policy directive aimed at controlling the growth of medical expenditures (51), emphasizing the need to curb excessive increases in healthcare costs. This policy may have had a delayed effect on resource allocation and efficiency in rural healthcare settings, potentially contributing to the observed decline in efficiency scores after 2018. While these external factors likely played a role in the declining efficiency trends, we acknowledge that internal inefficiencies within the primary healthcare system may have also contributed. Further research is needed to disentangle the specific contributions of external policy shifts and pandemic-related disruptions from structural inefficiencies in the healthcare system.

In this study, the MPI of village clinics across Jiangsu Province showed a general decline, primarily driven by a decrease in TC. TC plays a crucial role in resource allocation for village clinics, encompassing not only advancements in medical equipment but also the training and management of health technicians. The efficiency of village clinics is often constrained by slow technological progress, underscoring the need for technological innovation and application to enhance overall efficiency. Since 2018, TC has declined, likely due to macroeconomic pressures such as international trade tensions and the economic downturn caused by COVID-19. Additionally, healthcare cost-containment policies introduced in 2018 may have inadvertently restricted investment in medical technology (51).

### 4.2 Factors affecting resource allocation efficiency

#### 4.2.1 Population density

With rural urbanization, declining populations require a more tailored approach to primary healthcare. This study shows that population density is a significant factor influencing the resource allocation efficiency of village clinics. Higher population density is associated with more efficient healthcare resource allocation, as concentrated populations increase service demand, leading to more effective resource distribution (32, 52). In densely populated areas, village clinics benefit from economies of scale, reducing unit costs and improving service quality. Thus, policy planning should consider population density in clinic placement. In denser regions, expanding clinic numbers and service capacity may be beneficial, while in less populated areas, integrating and optimizing resources can improve efficiency. Regular assessments could help ensure that village clinics adapt to evolving healthcare needs.

#### 4.2.2 Per capita disposable income

As income increases, rural residents tend to change their demand structure for healthcare services. For most urban residents, rising incomes lead to increased demand for healthcare, disease prevention, and post-treatment services, as well as a preference for higher-level specialized services. Although there is still potential for growth in demand for healthcare services among low-income urban populations, for rural residents, higher income reduces concerns about impoverishment due to illness, transforming latent healthcare needs into visible, actual demands.

#### 4.2.3 Healthcare fiscal expenditure

Healthcare fiscal expenditure plays a crucial role in guiding the allocation of healthcare resources (42). Rural healthcare funding is primarily derived from fiscal expenditure, making economic growth a key factor in increasing government investment in rural healthcare. With adequate investment, the government, guided by the principle of health equity, should carefully consider both supply and demand sides when designing the structure of healthcare fiscal expenditure. This can reduce urban–rural disparities and improve the efficiency of service delivery by healthcare providers.

#### 4.2.4 Urbanization rate

As the process of urbanization accelerates, the urban population increases while the rural population declines, leading to a gradual reduction in the effective demand for rural healthcare services. In the absence of changes in current investment levels, this shift results in decreased efficiency in the supply of rural healthcare services. Furthermore, the reduction in the rural population leads to a gradual decrease in government investment in rural healthcare, which further negatively impacts the efficiency of healthcare service provision in rural areas.

### 4.3 Recommendations and measures to optimize resource allocation efficiency

#### 4.3.1 Improve healthcare investment policies

Adjust the supply structure and reasonably plan the quantity and scale of village-level medical resources in each city. This will optimize resource allocation by implementing differentiated policies for human, material, and financial investments across regions, ultimately improving effective supply. In cities with increasing returns to scale, efficiency can be enhanced by increasing investment and expanding scale. Conversely, in cities exhibiting decreasing returns to scale, the focus should be on reducing scale while maintaining output, thus minimizing investment. Based on the input–output redundancy analysis results from this study, for the corresponding cities, optimizing resource allocation efficiency could involve reducing the number of village clinics, cutting down on equipment and staff, and increasing medical revenue.

#### 4.3.2 Enhance the professional workforce in village clinics

Strengthen the professional workforce in village clinics by investing in medical technology and providing regular training to improve healthcare personnel’s skills. Establish medical alliances and dispatch senior hospital staff to village clinics for technical guidance. Additionally, support technical personnel from village clinics in further education at higher-level hospitals to enhance their ability to apply new technologies. This will improve service quality and increase patients’ trust in the medical capabilities of village clinics.

#### 4.3.3 Improve management and technological innovation at the village level

Enhance the organizational management and technological innovation of village clinics by actively exploring the application of information technology and telemedicine. Utilize shared medical data for decision-making and improve management capabilities (53).

## 5 Conclusion

This study analyzed the efficiency of healthcare resource allocation in village clinics in rural Jiangsu Province and identified key influencing factors. The results highlight low TE in village clinics, especially in DEA-inefficient cities where input–output ratios are imbalanced. TC is a critical factor affecting the MPI, emphasizing the need for greater investment in medical technology, enhanced training, and resource optimization to improve efficiency. The significant impact of population density on TE suggests that future policies should consider regional population variations to create more targeted healthcare service layouts, addressing the evolving needs of rural areas. These findings provide valuable insights for optimizing rural primary healthcare policies, promoting sustainable development of village clinics, and enhancing rural healthcare quality.

### 5.1 Limitation

This study has several limitations. Although we adopted bootstrapping DEA to correct bias, the dataset primarily relies on publicly available reports and administrative records. These sources, while authoritative, may not fully capture all aspects of healthcare resource allocation and utilization. In future studies, we will further refine the selection of indicators and consider integrating more advanced econometric models to improve the robustness of efficiency measurement and its influencing factors.

## Data Availability

The original contributions presented in the study are included in the article/Supplementary material, further inquiries can be directed to the corresponding authors.
