# Supplementary material for: Efficiency analysis of primary health care resources: DEA and Tobit regression evidence from village clinics in Jiangsu Province
Source: Front Public Health. 2025 Apr 23;13:1515532. doi: 10.3389/fpubh.2025.1515532 (PMC12055500; doi:10.3389/fpubh.2025.1515532)
Supplement: Supplementary file 1 [file Table_1.docx]

Supplementary TABLE 1 Sensitivity analysis

| Variables/removed | Efficiency average scores | Spearman rank correlation(sig) | Wilcoxon p value |
| --- | --- | --- | --- |
| Number of village clinics | 0.916 | 0.986(0.000) | 0.084 |
| Number of medical devices | 0.922 | 1.000(0.000) | 0.084 |
| Number of health technicians | 0.930 | 1.000(0.000) | >0.05 |
| Medical revenue | 0.924 | 0.885(0.000) | 0.317 |
