# Supplementary material for: Efficiency analysis of primary health care resources: DEA and Tobit regression evidence from village clinics in Jiangsu Province
Source: Front Public Health. 2025 Apr 23;13:1515532. doi: 10.3389/fpubh.2025.1515532 (PMC12055500; doi:10.3389/fpubh.2025.1515532)
Supplement: Supplementary file 2 [file Table_2.docx]

Supplementary TABLE 2 Temporal Stability Analysis

| Year | Spearman rank correlation(sig) | Wilcoxon p value |
| --- | --- | --- |
| 2015-2016 | 0.876 (0.000) | 0.299 |
| 2016-2017 | 0.545 (0.054) | 0.863 |
| 2017-2018 | 0.611(0.027) | 0.863 |
| 2018-2019 | 0.903 (0.000) | 0.047 |
| 2019-2020 | 0.975 (0.000) | 1.000 |
| 2020-2021 | 0.934 (0.000) | 0.931 |
| 2021-2022 | 0.631 (0.021) | 0.420 |
