# Supplementary material for: Efficiency analysis of primary health care resources: DEA and Tobit regression evidence from village clinics in Jiangsu Province
Source: Front Public Health. 2025 Apr 23;13:1515532. doi: 10.3389/fpubh.2025.1515532 (PMC12055500; doi:10.3389/fpubh.2025.1515532)
Supplement: Supplementary file 3 [file Table_3.docx]

Supplementary TABLE 3 Comparison of DEA and SFA results

| City | DEA | SFA | Spearman rank correlation(sig) | Wilcoxon p value |
| --- | --- | --- | --- | --- |
| Nanjing | 0.662 | 0.858 | 0.621 (0.023) | 0.807 |
| Wuxi | 1 | 0.957 |  |  |
| Xuzhou | 0.904 | 0.936 |  |  |
| Changzhou | 1 | 0.999 |  |  |
| Suzhou | 1 | 0.945 |  |  |
| Nantong | 1 | 0.858 |  |  |
| Lianyungang | 1 | 0.990 |  |  |
| Huaian | 0.698 | 0.807 |  |  |
| Yancheng | 0.795 | 0.816 |  |  |
| Yangzhou | 0.514 | 0.725 |  |  |
| Zhenjiang | 1 | 0.806 |  |  |
| Taizhou | 0.741 | 0.718 |  |  |
| Suqian | 0.978 | 0.881 |  |  |
